# Catheter Ablation versus Thoracoscopic Surgical Ablation in Long Standing Persistent Atrial Fibrillation (CASA-AF): study protocol for a randomised controlled trial

**DOI:** 10.1186/s13063-018-2487-9

**Published:** 2018-02-20

**Authors:** Habib Rehman Khan, Ines Kralj-Hans, Shouvik Haldar, Toufan Bahrami, Jonathan Clague, Anthony De Souza, Darrel Francis, Wajid Hussain, Julian Jarman, David Gareth Jones, Neeraj Mediratta, Raad Mohiaddin, Tushar Salukhe, Simon Jones, Joanne Lord, Caroline Murphy, Joanna Kelly, Vias Markides, Dhiraj Gupta, Tom Wong

**Affiliations:** 1 Royal Brompton and Harefield NHS Trust, London, UK; 2 National Heart and Lung Institute, Imperial College London, London, UK; 3 Institute of Cardiovascular Medicine and Science, London, UK; 4 Liverpool Heart and Chest Hospital NHS Trust, Liverpool, UK; 5 New York University School of Medicine, New York, NY USA; 6 Southampton Health Technology Assessments Centre (SHTAC), University of Southampton, Southampton, UK; 7 King’s Clinical Trials Unit, Institute of Psychiatry, King’s College London, London, UK; 8 Royal Brompton Hospital, Sydney Street, London, UK

**Keywords:** Long-standing persistent atrial fibrillation, Catheter ablation, Thoracoscopic surgical ablation, Randomised clinical trial, Implantable loop recorder, Left atrial appendage exclusion

## Abstract

**Background:**

Atrial fibrillation is the commonest arrhythmia which raises the risk of heart failure, thromboembolic stroke, morbidity and death. Pharmacological treatments of this condition are focused on heart rate control, rhythm control and reduction in risk of stroke. Selective ablation of cardiac tissues resulting in isolation of areas causing atrial fibrillation is another treatment strategy which can be delivered by two minimally invasive interventions: percutaneous catheter ablation and thoracoscopic surgical ablation. The main purpose of this trial is to compare the effectiveness and safety of these two interventions.

**Methods/design:**

Catheter Ablation versus Thoracoscopic Surgical Ablation in Long Standing Persistent Atrial Fibrillation (CASA-AF) is a prospective, multi-centre, randomised controlled trial within three NHS tertiary cardiovascular centres specialising in treatment of atrial fibrillation. Eligible adults (*n* = 120) with symptomatic, long-standing, persistent atrial fibrillation will be randomly allocated to either catheter ablation or thoracoscopic ablation in a 1:1 ratio. Pre-determined lesion sets will be delivered in each treatment arm with confirmation of appropriate conduction block. All patients will have an implantable loop recorder (ILR) inserted subcutaneously immediately following ablation to enable continuous heart rhythm monitoring for at least 12 months. The devices will be programmed to detect episodes of atrial fibrillation and atrial tachycardia ≥ 30 s in duration. The patients will be followed for 12 months, completing appropriate clinical assessments and questionnaires every 3 months. The ILR data will be wirelessly transmitted daily and evaluated every month for the duration of the follow-up. The primary endpoint in the study is freedom from atrial fibrillation and atrial tachycardia at the end of the follow-up period.

**Discussion:**

The CASA-AF Trial is a National Institute for Health Research-funded study that will provide first-class evidence on the comparative efficacy, safety and cost-effectiveness of thoracoscopic surgical ablation and conventional percutaneous catheter ablation for long-standing persistent atrial fibrillation. In addition, the results of the trial will provide information on the effects on patients’ quality of life.

**Trial registration:**

ISRCTN Registry, ISRCTN18250790. Registered on 24 April 2015.

**Electronic supplementary material:**

The online version of this article (10.1186/s13063-018-2487-9) contains supplementary material, which is available to authorized users.

## Background

Atrial fibrillation (AF) is the most common arrhythmia, characterised by an irregularly irregular pulse, loss of atrial contractile function and attendant loss of active ventricular filling. It is an important public health concern because it affects 1–2% of the population. Furthermore, its prevalence is on the increase and is likely to continue to rise with an aging population [1–5]. AF is associated with lower quality of life, increased morbidity (5-fold increase in thromboembolic stroke, 3-fold increase in heart failure) and mortality (2-fold increased fatality) and results in a large number of hospital admissions [4–8]. AF treatment is usually lifelong and costly, resulting in considerable burden on national health resources. Some estimates suggest that AF accounts for ~ 2% of the NHS budget in the United Kingdom [2, 8–10].

Stroke prevention with anticoagulants is the cornerstone in management of patients with AF. Additionally, pharmacological agents for heart rhythm or rate control may be used, depending on clinical indications and patient characteristics. Rhythm control is preferred in patients with persistent AF who continue to experience limiting symptoms despite adequate rate control [2, 5, 11, 12].

Traditionally, rhythm control is attempted with anti-arrhythmic drugs (AADs) and/or direct current (DC) cardioversion, but the long-term efficacy of this approach is poor and is associated with drug side effects and risk of pro-arrhythmia. Percutaneous catheter ablation has been shown to be more effective than AAD therapy in achieving and maintaining normal sinus rhythm (SR) [4, 5, 11–18] and is now routinely offered as standard management of patients with symptomatic AF [2, 5, 12].

Conventional catheter ablation can reliably maintain SR in a large proportion of patients with paroxysmal AF (up to 78%) [8, 12, 19–22] and in a majority of patients with short-lasting persistent AF [23–27]. However, its success rate in maintaining SR in patients with long-standing persistent atrial fibrillation (LSPAF) is poor at only 32–40%, with frequent requirement of more than one procedure to increase success rates [8, 28–32].

Minimally invasive thoracoscopic surgical ablation provides another option for treatment of AF, and there are indications that it may be more effective than catheter ablation in restoring SR due to direct access and ability to execute transmural and contiguous lesions, ganglionic plexi ablation and ability to excise left atrial appendage (LAA) [33–36]. Any surgical procedure carries a risk of morbidity and complications, but risks associated with thoracoscopic surgical ablation have not been directly compared with catheter ablation.

In this trial, we will compare the effectiveness of the two procedures in maintaining freedom from arrhythmia in patients with LSPAF, with the associated effect on quality of life. We will also examine the complications associated with each ablation technique, as well as their cost-effectiveness.

## Methods/design

CASA-AF is a prospective, multicentre, randomised clinical trial designed to assess the effectiveness, safety and cost-effectiveness of thoracoscopic surgical ablation compared with catheter ablation (usual care) in patients with LSPAF. It is on-going within three tertiary specialist NHS hospitals in England with proven expertise in both interventions and a large pool of potentially eligible participants (Royal Brompton Hospital, Harefield Hospital, and Liverpool Heart and Chest Hospital).

**Fig. 1:**
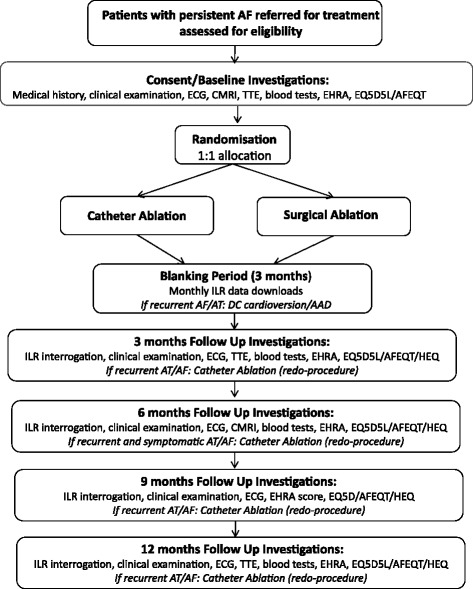
Schematic representation of Catheter Ablation versus Thoracoscopic Surgical Ablation in Long Standing Persistent Atrial Fibrillation (CASA-AF) study design. *AAD* Anti-arrhythmic drug, *AF* Atrial fibrillation, *AFEQT* Atrial Fibrillation Effect on QualiTy-of-Life Questionnaire, *AT* Atrial tachycardia, *cMRI* Cardiac magnetic resonance imaging, *DC* Direct current, *ECG* Electrocardiogram, *EHRA* European Heart Rhythm Association, *EQ-5D-5L* EuroQol standardised health questionnaire, *HEQ* Health economics questionnaire, *ILR* Implantable loop recorder, *TTE* Transthoracic echocardiogram

**Fig. 2:**
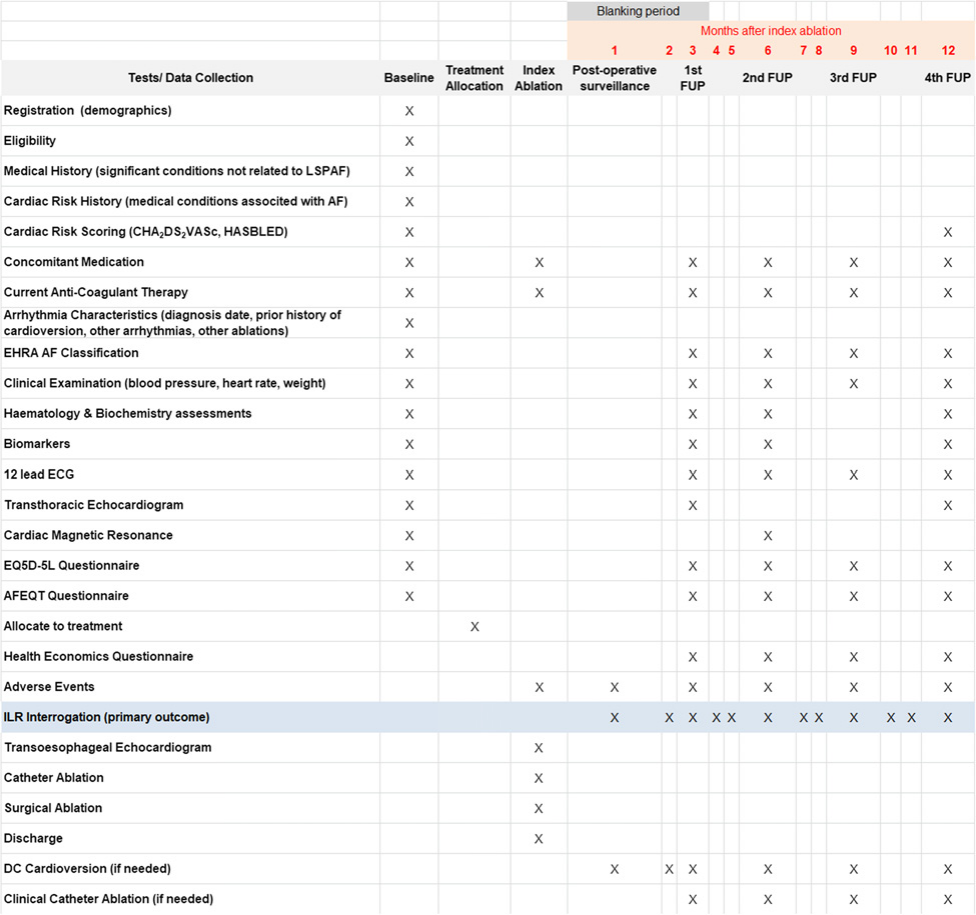
Schedule of enrolment, tests and assessments in the Catheter Ablation versus Thoracoscopic Surgical Ablation in Long Standing Persistent Atrial Fibrillation (CASA-AF) study (Standard Protocol Items: Recommendations for Interventional Trials [SPIRIT] figure). *AF* Atrial fibrillation, *AFEQT* Atrial Fibrillation Effect on QualiTy-of-Life Questionnaire, *CHA*_*2*_*DS*_*2*_*VASc score* Congestive heart failure (or left ventricular systolic dysfunction), hypertension (blood pressure consistently > 140/90 mmHg or treated hypertension on medication), age ≥ 75 years, diabetes mellitus, prior stroke or transient ischaemic attack or thromboembolism, vascular disease (e.g., peripheral artery disease, myocardial infarction, aortic plaque), age 65–74 years, sex category (i.e., female sex), *DC* Direct current, *EHRA* European Heart Rhythm Association, EQ-5D-5 L EuroQol standardised health questionnaire, *FUP* Follow-up, *HAS-BLED score* Hypertension, abnormal renal/liver function, stroke, bleeding history or predisposition, labile international normalised ratio, elderly, drugs/alcohol concomitantly, *ILR* Implantable loop recorder, *LSPAF* Long-standing persistent atrial fibrillation

This protocol follows the guidance of Standard Protocol Items: Recommendations for Interventional Trials (SPIRIT) 2013 statement [37, 38], and it includes the schedule of enrolment and relevant assessments (Figs. 1 and 2) based on the SPIRIT figure template. A completed SPIRIT checklist is provided in Additional file 1.

### Outcomes of the study

#### Primary

The main objective of this trial is to identify the most effective ablative intervention for treating LSPAF. The primary hypothesis is that thoracoscopic surgical ablation is more effective than percutaneous catheter ablation in achieving freedom from atrial arrhythmia. The primary outcome in the study is therefore freedom from arrhythmia after a single ablation procedure and without AADs during study follow-up (starting from the end of the blanking period of 3 months, up to 12 months after the ablation). Data collected by the implantable loop recorder (ILR) over the follow-up period will be used to detect the recurrence of AF/atrial tachycardia (AF/AT) ≥ 30 s.

#### Secondary

The most important among the secondary outcomes is the safety of the two interventions. The safety endpoint in this trial is intervention-related major complication, defined as permanent injury or death, one that requires unplanned intervention for treatment, or one that prolongs or requires unplanned hospitalisation for > 48 h. In addition, we will evaluate the following:

- Secondary efficacy outcome, defined as freedom from atrial arrhythmias following multiple procedures without AADs during 12 months of follow-up
- The clinical success of the two interventions by comparing reduction of AF burden (≥ 75%) over the follow-up period in each treatment arm
- Changes in atrial size and function following ablation using echocardiography and cardiac magnetic resonance imaging (cMRI) parameters
- The effects of arrhythmia interventions on patients’ symptoms and quality of life through changes in European Heart Rhythm Association (EHRA), EuroQol standardised health questionnaire (EQ-5D-5L) and Atrial Fibrillation Effect on QualiTy-of-Life Questionnaire (AFEQT) scores [39, 40]
- Quality-adjusted life-years (QALYs) accrued during the 12-month study period
- Cost-effectiveness (incremental cost per QALY gained) for surgical ablation compared with catheter ablation estimated over the 12-month study period (‘within-trial’ analysis) and over a lifetime horizon (estimated by modelling)

### Characteristics of participants

Adults with symptomatic LSPAF considered for treatment (DC cardioversion or catheter ablation) at the participating trial centres will be considered for inclusion in the study. The complete list of inclusion and exclusion criteria is presented in Table 1.

**Table 1:** Eligibility criteria in Catheter Ablation versus Thoracoscopic Surgical Ablation in Long Standing Persistent Atrial Fibrillation trial

*Inclusion criteria*
Age ≥ 18 years
Long-standing persistent AF (> 12 months’ duration)
EHRA symptom score > 2 (*see* Additional file 1)
Left ventricular ejection fraction ≥ 40%
Suitable for either ablation procedure
*Exclusion criteria*
Valvular heart disease with severity greater than mild
Contraindication to anticoagulation
Thrombus in the left atrium despite anticoagulation in therapeutic range
Cerebrovascular accident within the previous 6 months
Previous thoracic or cardiac surgery (including surgical interventions for AF)
Prior left atrial catheter ablation for AF
Unable to provide informed written consent
Active malignancy, another severe concomitant condition or presence of implanted cardiac devices that would preclude patient undergoing study-specific procedures
Pregnant or breastfeeding, or women of childbearing age not using a reliable contraceptive method

*AF* Atrial fibrillation, *EHRA* European Heart Rhythm Association

The consultant cardiologists at participating sites will refer patients they identify in the outpatient clinics or through DC cardioversion and ablation waiting lists for inclusion in the trial. The aim is to continue screening until the required number of participants (*n* = 120) has been allocated to treatment. Patients can withdraw from the study at any time in the follow-up period and will not be replaced, because levels of attrition have formed part of the sample size calculation.

### CASA-AF trial schedule

Patients considered eligible to take part in the study will be provided with a participant information sheet and given opportunities to discuss details of potential participation with the study team. They will be encouraged to discuss the information with their family, friends and general practitioners before making a decision to participate in the trial. When they confirm their interest in taking part, a hospital appointment will be arranged for them to sign an informed consent form and to complete baseline assessments. Participants’ progress through the study is shown schematically in Fig. 1.

### Baseline study visit

As part of the baseline visit, we will collect blood samples for routine haematology, biochemistry and coagulation assessments as well as samples for cardiac biomarker evaluation. With patients’ permission, surplus samples will be stored for future research by biobanks at the participating centres (Cardiovascular Biomedical Research Unit Biobank at the Royal Brompton and Harefield NHS Trust and the Liverpool Bio-Innovation Hub Biobank associated with Liverpool Heart and Chest Hospital NHS Trust).

Routine haematology and biochemistry assessments include full blood count, electrolytes, renal function tests, coagulation profile, liver function tests, thyroid function tests, C-reactive protein, tests for diabetes (haemoglobin A1C) and lipid profile (high-density lipoprotein, low-density lipoprotein). Transthoracic echocardiogram (TTE) and cMRI assessment will ensure that patients do not have poor left ventricular (LV) function, valvular disease or other pathologies that meet the exclusion criteria. Additional clinical and study data collected at baseline, as well as at other study time points, are shown in Fig. 2 (SPIRIT figure). A detailed study codebook contains a complete list of all the variables, including type of data and reference values where appropriate.

#### Treatment allocation

Patients with confirmed eligibility for participation at baseline assessments will proceed to being assigned to treatment group by computer-generated sequence in a 1:1 ratio using minimisation. A secure 24-h bespoke web-based system hosted by the King’s Clinical Trials Unit provides named research team members with access to the allocation system where they enter each participant’s unique participant identification number, initials and date of birth in an electronic online form. In addition, the system uses the study site code, participant’s sex and the size of the left atrium (< 50 mm, > 50 mm) as stratifying variables. Allocation is concealed, but blinding is not possible in this trial. However, the primary outcome assessor will be blinded to treatment arms.

#### Pre-ablation

##### Anticoagulation

According to current guidelines [2, 9, 41–44], it is recommended that patients randomised to catheter ablation remain on uninterrupted warfarin treatment unless a different strategy is outlined by the operator. If patients are treated with novel oral anticoagulants (NOACs), they can be converted to warfarin treatment for 4 weeks before the catheter ablation procedure, continue with NOACs or stop the therapy 24–36 h before the ablation procedure, depending on the type of NOAC and clinician’s preference. For patients randomised to surgical ablation, it is current practice to stop warfarin therapy 5 days before the procedure and have anticoagulation bridging with enoxaparin at a dose of 1.5 mg/kg once daily for 3 days with no anticoagulation 1 day before they have the procedure.

NOAC treatment will also be discontinued for patients randomised to surgical ablation. The timing of discontinuation will depend on the type of drug and renal function, but in general NOACs will be stopped 2–5 days before surgery. Anticoagulation protocols may be altered as guidelines are updated and local practices change.

#### Ablation procedures

##### Thoracoscopic surgical AF ablation

Details of the operation have been described previously by Yilmaz and others [45–47]. In this trial the protocol additionally mandates the presence of a cardiac electrophysiologist in theatre during surgical ablation to ensure that conduction block is tested and achieved for all lesions, because this has been associated with a trend towards better outcomes [48]. Cardiac surgeons participating in this study need to be experienced in video-assisted thoracoscopic surgery and to have conducted at least 20 thoracoscopic ablations for AF as the primary operator.

Transoesophageal echocardiography (TOE) will be performed with the patient under general anaesthesia to exclude left atrial (LA) thrombus prior to the start of the procedure. Three thoracoports will be introduced on each side. Pulmonary veins (PVs) and LA antrum will be exposed by pericardiotomy and blunt dissection using a Lumitip™ dissector (AtriCure, Inc., West Chester, OH, USA) into the transverse and oblique sinuses. Pulmonary vein isolation (PVI) will be performed from the epicardial surface using a bipolar radiofrequency ablation clamp (AtriCure, Inc.) and overlapping applications around the LA antrum. PVI will be confirmed at the end of the procedure by testing for entrance and exit block during pacing on both sides of the lesion in SR. Further ablation will be performed if PVI is incomplete. An Isolator multifunctional pen (AtriCure) will then be used to ablate ganglionic plexi located by high-frequency stimulation. Additional linear lines will be undertaken using the multifunctional pen (AtriCure) connecting the contralateral superior PVs (roof line) and the inferior PVs (inferior line) to create a posterior wall box lesion. If AF persists, the SR will be restored by external DC cardioversion. Sensing and pacing manoeuvres will then be used to verify electrical isolation of the posterior box in SR. The LAA will then be excluded using the AtriClip® LAA excluder system (AtriCure) owing to recent emerging evidence of improved outcomes with LAA exclusion mechanically and electrical isolation [49–52]. The ILR will be implanted at the end of the procedure, and patients will be extubated in the operating theatre.

##### Catheter AF ablation (usual care treatment)

TOE will exclude LA thrombus under general anaesthesia and guide trans-septal puncture. Patients will be heparinised to maintain an activated clotting time (ACT) between 300 and 350 s. The CARTO 3 three-dimensional electroanatomical mapping system (Biosense Webster, Irvine, CA, USA) will be used to create the LA geometry with a 20-pole circular mapping catheter (LASSO 2515 NAV; Biosense Webster). Simultaneous voltage maps will be created using the CARTO® CONFIDENSE™ software module. The voltage maps will be created in AF and in SR once the patient is cardioverted. This will allow for atrial low-voltage comparison to LA fibrosis captured by cMRI. Ablation will be conducted with THERMACOOL® SMARTTOUCH™ 3.5-mm irrigated-tip catheter (Biosense Webster). A stepwise ablation strategy will be used to electrically isolate the PVs at the antral level, then linear ablation will be performed at the LA roof along with creation of a posterior line (to create a ‘box lesion’). A lateral mitral isthmus line will be performed, and, finally, ablation at the cavotricuspid isthmus ablation will be performed. Electrical isolation of the PVs will be confirmed through testing for both entrance and exit blocks with the circular sensing catheter. The integrity of the linear lesions will be assessed by differential pacing manoeuvres. If block is not achieved, further ablation will be performed to achieve bi-directional block across the linear lesions. If AT occurs at any point, it will be mapped and ablated when possible [53]. An ILR will be implanted at the end of the procedure once the ACT has normalised following heparin reversal with intravenous protamine. Patients will then be extubated in the cardiac catheterisation laboratories.

#### Post-operative management

The patients will be managed post-operatively according to standardised hospital protocols described briefly below.

##### Post-operative analgesia

Participants in the surgical ablation arm will receive intercostal nerve block at each port site (bupivacaine or similar agents; dosage depending on patient characteristics and tolerance), paracetamol (1 g four times daily intravenously or per rectum and then by mouth) and codeine (30–60 mg up to four times daily). Patients can also be given tramadol (50 mg intramuscularly) if appropriate. In the first 24 h post-procedure the patient will be receiving patient-controlled analgesia with fentanyl or morphine or other opioids, depending on the anaesthetist’s recommendations and current clinical practice. Opiates should be reduced 12 h post-operatively.

One day after surgery, if patients have normal renal function and are able to tolerate oral medication, analgesia could be provided by non-steroidal anti-inflammatory drugs for 1 week with or without opiates. On discharge, the patients will have a supply of analgesics for 28 days to be taken as needed. Participants in the catheter ablation arm will be treated with paracetamol and codeine as required.

##### Post-operative anticoagulation

After a minimum of 3 h following ablation, patients will receive anticoagulation if there are no contraindications. Anticoagulation in the surgical group will be achieved by using enoxaparin (1.5 mg/kg once daily) or other heparin derivatives to ensure rapid reversal if needed. Once chest drains are removed, the patient’s usual anticoagulation (warfarin or NOAC) will be restarted. Anticoagulation in the catheter group will continue uninterrupted in normal routine. Anticoagulation therapy will continue for the duration of the trial follow-up.

##### Post-operative AADs

AAD therapy (flecainide, procainamide, amiodarone or sotalol) will continue for a maximum of 3 months (blanking period) after the ablation procedure, at which point it should be terminated. Drugs that may have an effect on heart rhythm but are used to treat other conditions such as hypertension will continue to be administered as clinically indicated (i.e., β-blockers and non-dihydropyridine calcium channel blockers).

##### Post-operative antibiotic prophylaxis

In both treatment arms patients will usually receive one dose of antibiotic on induction of anaesthesia, one dose at the end of the procedure, and usual dose individual antibiotics for 5 days post-operatively, depending on local practices.

##### Early post-operative discharge period

Following discharge, study participants will be contacted by the research team once per week for the first month to assess their health status. They will be asked about pain management, cough, raised temperature, difficulties swallowing and any other symptoms that may be early indications of possible complications. In addition, patients will be advised to contact the study team if they have any concerns regarding their health for the duration of the study. If deemed appropriate the patients will be brought back to the study centre for full assessment, evaluation and treatment of health issues, even if the likelihood of those issues being related to study procedures is remote.

##### Implanted loop recorder

The Medtronic Reveal LINQ ILR (Medtronic, Minneapolis, MN, USA) will be used to monitor and assess patients’ heart rhythm. The device is highly sensitive and programmable to detect the occurrence of AF and AT lasting ≥ 30 s [54, 55]. Programming parameters of the device are summarised in Table 2.

**Table 2:** Programming parameters for Reveal LINQ™ implantable loop recorder

Programming feature	Parameter programmed	Duration
Indication	After AF ablation	
AT/AF	Both ‘on’	
AT	Rates ≥ 100 /minutes	
Tachycardia	> 162 /minutes	48 beats
Asystole	4.5 s	
Bradycardia	< 40/min	8 beats
Patient symptom capture	7.5 min	4 episodes

*AF* Atrial fibrillation, *AT* Atrial tachycardia

The patients are registered on the Medtronic CareLink® Network following ILR insertion and instructed on how to perform manual data downloads using their home monitoring equipment. Data from the ILRs are uploaded to the CareLink® server over mobile phone networks. Patients are asked to perform manual data downloads once per week or more frequently, depending on the burden of collected data in individual cases.

Named study team members are granted access to the CareLink® Network server to monitor ILR data uploads for their patients. A dedicated cardiac physiologist, blinded to treatment allocation, analyses downloaded data on a regular basis to produce monthly heart rhythm assessment reports for each patient. A panel of three experts is nominated to adjudicate in cases where heart rhythm is assessed as ambiguous by the cardiac physiologist.

#### Follow-up schedule

Details of the assessments during study participation are given in Fig. 2 as per SPIRIT 2013 recommendations. Recurrence of symptomatic AF during the blanking period (up to 3 months following ablation) will be treated with DC cardioversion with or without use of AADs, depending on patient tolerance and comorbidities. Patients will be offered percutaneous catheter ablation if the AF recurs at a later point in the follow-up period as shown in Fig. 1.

### Data

#### Data collection and management

Source data worksheets and electronic case report forms (eCRFs) have been designed with input from the chief investigator (CI), principal investigator, study statistician and relevant co-applicants. Paper source data worksheets will be used for each patient to collect appropriate study data, and these will then be transcribed into the eCRF database. Trained individuals at each trial centre are named on the delegation of duties log as responsible for data collection and entry on the eCRF. Source data worksheets will be reconciled at the end of the trial with patients’ NHS medical notes in the recruiting centre.

The InferMed MACRO® database system (Elsevier, Amsterdam, the Netherlands) was used to create the eCRF, and it will be used to manage anonymised study data. It has programmable referential data rules and range checks to ensure data integrity at the time of data entry. Any data edits can be monitored through database audit trails, and the security is enforced by individual logins and passwords. This system is hosted and maintained on a secure dedicated server by the King’s Clinical Trials Unit.

#### Adverse events/serious adverse events

All adverse events (AEs) occurring during the course of the trial will be collected, documented and recorded by the research team at each trial centre. Patients are advised to contact the research team if they have any health concerns during the study follow-up and will be prompted to report those at every study visit.

The research teams will ensure that known complications as well as unexpected events/reactions are detailed in patients’ study files as well as their medical notes. The teams will follow an agreed protocol for timely reporting of serious adverse events (SAEs), which involves written notification to the chief investigator and the study sponsor as soon as possible. A list of expected complications of the ablative procedures is shown in Table 3.

**Table 3:** Known complications associated with ablative procedures in Catheter Ablation versus Thoracoscopic Surgical Ablation in Long Standing Persistent Atrial Fibrillation study

Adverse events	Serious adverse events
Bruising, hematoma, vascular injury not requiring intervention	Vascular complications requiring blood transfusion or intervention
Pericardial/pleural effusion (observation only)	Symptomatic pericardial/pleural effusion or requiring intervention
Broken rib	Stroke/transient ischemic attack
Pneumothorax requiring observation	Pneumothorax requiring chest drain
Infection (i.e., pneumonia)	Empyema
Pulmonary oedema	Myocardial infarction
Temporary phrenic nerve damage	Permanent phrenic nerve damage
Pain near surgical sites	Pulmonary vein stenosis (> 50% reduction in diameter from baseline)
	Requirement to insert PPM (with or without prior conduction tissue damage)
	Cardiac trauma requiring surgical intervention
	Radiation-induced skin damage
	Oesophageal atrial fistula
	Death

*PPM* Permanent pacemaker

Recurrence of AF and subsequent hospital admission for DC cardioversion or percutaneous catheter ablation will not be treated as SAEs in the trial, but will be recorded and reported with the results.

### Data from imaging modalities and questionnaires

cMRI will be performed during the baseline visit and again at 6 months after the ablation. It will be used to capture LA area and length [56, 57] and to calculate maximum and minimum LA volumes as well as the cross-sectional diameter of PVs. The images will be acquired using planes in the LV long- and short-axis stacks. A specific protocol of sequences for late gadolinium enhancement (LGE) of the LA will be used to evaluate location and quantification of LA scars [58]. The LV function will also be measured by volume quantification in systole and diastole using endocardial tracing [59–61].

TTEs will be performed at baseline, 3 months after the ablation and at the last study visit (12 months after the ablation). In addition to the standard clinical protocol set out by British Society of Echocardiography, we will acquire images to allow for offline assessment of LA function (measurement of myocardial velocities, LA strain and strain rate) by using tissue Doppler and 2D speckle-tracking methods [62–64].

The EHRA symptoms score and AFEQT and EQ-5D-5L questionnaires will be used at each study visit to assess any changes in patients’ symptoms and quality of life from baseline to the end of the follow-up period [39, 65, 66]. We have also designed a health economics questionnaire to collect patient-reported data on the use of primary, secondary and community health and social care services following ablation to estimate costs.

### Monitoring

An independent data monitoring committee (DMC) consisting of four members (two cardiologists, a surgeon and a statistician) has been established, with functions and responsibilities detailed in a DMC charter. The DMC meets at least twice annually and communicates their advice to the trial steering committee (TSC).

The TSC has executive power and consists of seven members, five of whom are independent (two cardiologists, one surgeon and two lay members). This committee will meet twice annually for the duration of active patient recruitment and follow-up, and additional ad hoc meetings may also be scheduled if necessary. This group’s functions and remit are defined in the TSC charter. Two external independent groups of experts specialising in electrophysiology, cardiology and thoracic surgery will be formed to evaluate primary and safety endpoints of the trial.

### Statistical analyses

All statistical analyses will be based on intention to treat, and the data analysed will be derived from all randomised participants. We will also perform sensitivity analyses to explore the impact of missing data, non-compliance and withdrawals. R statistical software version 3.0.2 (or later; R Foundation for Statistical Computing, Vienna, Austria) will be used to analyse data.

Sample size (*n* = 120) was calculated on the basis of data derived from our pilot study showing freedom for AF/AT at 6 months in the surgical group in 76% versus 44% in the catheter group. Using these results a sample size of 48 per group will be required to detect a difference in the primary outcome with 90% power and a 5% significance level. There is a margin of error of 25%, which includes a 10% dropout rate.

The primary outcome of the trial is the proportion of patients with LSPAF undergoing ablation who are free from atrial arrhythmias (defined as a single episode of ≥ 30 s) within 1 year after a single ablation procedure. ‘Arrhythmia-free’ patients will be identified through ILR data assessments performed monthly by a single blinded cardiac physiologist. Chi-square tests will be used for comparisons between the trial arms. A logistic regression model will be developed to estimate the probability of being free from AF at 1 year by either procedure. The primary measure to be reported is the OR of being ‘AF-free’ for the surgical group after the other factors in the model have been controlled for. The recurrence of AF and duration of AF freedom will be analysed using Kaplan-Meier survival curves.

Binary secondary outcomes (reduction in arrhythmia burden, freedom from arrhythmia following multiple procedures) will be analysed in the same manner as the primary outcome using a combination of chi-square tests and logistic regression. Freedom from arrhythmia following multiple procedures will be analysed using Kaplan-Meier survival curves.

Continuous data will be analysed using either Student’s *t* test or the Mann-Whitney *U* test and presented as mean ± SD, mean (95% CI) or median (IQR), depending upon distribution of obtained data. *P* < 0.05 will be considered significant.

### Health economic analysis

Cost-effectiveness will be assessed using both trial-based and model-based health economic analyses. Both will follow international methodological guidelines [67–70] and the ‘reference case’ recommended by the National Institute for Health and Care Excellence (NICE) for use in its technology appraisals [71–74], including the use of an NHS and personal social services perspective for costing and discounting of costs and QALYs at an annual rate of 3.5%.

In the trial-based analysis we will use EQ-5D-5L and health and social care resource use data to estimate the costs and QALYs accrued over the 12-month follow-up period by trial participants. In our main analysis we will include costs for all health and social care recorded in the eCRF and reported by patients in the health economics questionnaire at 3, 6, 9 and 12 months. The unit costs of services will be based on national average estimates from published sources [75–78]. We will also conduct a sensitivity analysis including only costs judged by the research team to be potentially related to AF or to AF treatment. QALYs will be estimated from survival data and quality of life (EQ-5D-5L) scores at 0, 3, 6, 9 and 12 months using an AUC approach. EQ-5D-5L scores will be calculated using the 2016 value set for England [79]. Mean between-group differences in QALYs and costs will be estimated using a bivariate regression approach, taking account of correlations between costs and effects and adjusting for any baseline differences in EQ-5D-5L scores or other key patient characteristics (such as age, CHA_2_DS_2_VASc score [congestive heart failure {or left ventricular systolic dysfunction}, hypertension {blood pressure consistently > 140/90 mmHg or treated hypertension on medication}, age ≥ 75 years, diabetes mellitus, prior stroke or transient ischaemic attack or thromboembolism, vascular disease {e.g., peripheral artery disease, myocardial infarction, aortic plaque}, age 65–74 years, sex category {i.e., female sex}] or HAS-BLED score [hypertension, abnormal renal/liver function, stroke, bleeding history or predisposition, labile international normalised ratio, elderly, drugs/alcohol concomitantly]). Multiple imputations will be used to account for missing data if appropriate [39, 65, 69, 80]. If the results indicate a trade-off between costs and health effects, an incremental cost-effectiveness ratio will be calculated—the ‘cost per QALY’. The extent of uncertainty over the results will be estimated using bootstrap regression and presented in the form of a cost-effectiveness acceptability curve [81].

A model-based economic analysis will also be conducted to estimate long-term benefits, harms and costs of surgical and catheter ablation compared with AAD therapy in patients with LSPAF. This will extrapolate costs and health outcomes observed in the trial, including freedom from arrhythmia, utility (EQ-5D-5L scores) and incidence of major side effects, over a long time horizon (up to lifetime). The model will also allow us to estimate costs and outcomes for the trial participants under medical management, which will provide further information about the comparative cost-effectiveness of treatment options for this patient group for healthcare commissioners and research funders. The model will be based on the MAPGuide AF model [82]. This is a discrete event simulation which estimates lifetime costs and QALYs for a heterogeneous population of individuals with AF treated according to a defined pathway of care, including anti-thrombotic and AAD therapy. The base case version of the model reflects the recommended care pathway in the NICE clinical guideline for AF. This care pathway can be changed to estimate costs and QALYs associated with different treatments (e.g., catheter ablation, thoracoscopic surgical ablation or AAD).

## Discussion

The use of traditional pharmacological agents to treat LSPAF is not satisfactory, owing to their serious side effects and need for lifelong treatment. Catheter ablation is widely accepted and effective therapy for paroxysmal AF, but its effectiveness in patients with LSPAF is limited, and multiple ablations may be required to relieve patients of symptoms [83–86]. On the basis of the encouraging results of thoracoscopic surgical ablation in several cohort studies of subjects with persistent AF and LSPAF and our pilot study [45, 87, 88], we have designed this trial to compare the two ablative treatments and provide first-class evidence of their efficacy, safety and cost-effectiveness.

## Trial status

The trial was open to recruitment on 1 August 2015 and currently is actively recruiting at two three NHS specialist care centres (Royal Brompton and Harefield NHS Foundation Trust, Brighton and Sussex University Hospitals NHS Trust and Liverpool Heart and Chest NHS Foundation Trust). Ashford and St Peter’s Hospitals NHS Trust is also registered as a patient identification centre.

## Additional file

Additional file 1: SPIRIT checklist. (DOC 120 kb)

## Abbreviations

AAD: Anti-arrhythmic drug
ACT: Activated clotting time
AE: Adverse event
AF: Atrial fibrillation
AFEQT: Atrial Fibrillation Effect on QualiTy-of-Life Questionnaire
AT: Atrial tachycardia
CASA-AF: Catheter Ablation versus Thoracoscopic Surgical Ablation in Long Standing Persistent Atrial Fibrillation
cMRI: Cardiac magnetic resonance imaging
DC: Direct current
DMC: Data monitoring committee
ECG: Electrocardiogram
eCRF: Electronic case report form
EHRA: European Heart Rhythm Association
EQ-5D-5L: EuroQol standardised health questionnaire
FUP: Follow-up
HEQ: Health economics questionnaire
ILR: Implantable loop recorder
LA: Left atrial
LAA: Left atrial appendage
LGE: Late gadolinium enhancement
LSPAF: Long-standing persistent atrial fibrillation
LV: Left ventricular
MRC: Medical Research Council
NICE: National Institute for Health and Care Excellence
NIHR: National Institute for Health Research
NOAC: Novel oral anticoagulant
PPM: Permanent pacemaker
PV: Pulmonary vein
PVI: Pulmonary vein isolation
QALY: Quality-adjusted life-year
SAE: Serious adverse event
SPIRIT: Standard Protocol Items: Recommendations for Interventional Trials
SR: Sinus rhythm
TOE: Transoesophageal echocardiography
TSC: Trial steering committee
TTE: Transthoracic echocardiogram
